# Synchronised infection identifies early rate-limiting steps in the hepatitis B virus life cycle

**DOI:** 10.1111/cmi.13250

**Published:** 2020-09-28

**Authors:** Anindita Chakraborty, Chunkyu Ko, Christin Henning, Aaron Lucko, James M. Harris, Fuwang Chen, Xiaodong Zhuang, Jochen M. Wettengel, Stephanie Roessler, Ulrike Protzer, Jane A. McKeating

**Affiliations:** 1 Institute of Virology, Technical University of Munich, School of Medicine/Helmholtz Zentrum München, Munich, Germany; 2 Technical University of Munich, Institute for Advanced Study, Munich, Germany; 3 Nuffield Department of Medicine, University of Oxford, Oxford, UK; 4 Institute of Pathology, University Hospital Heidelberg, Heidelberg, Germany; 5 German Center for Infection Research (DZIF), Munich, Germany

**Keywords:** hepatitis B, kinetics, virus internalisation

## Abstract

Hepatitis B virus (HBV) is an enveloped DNA virus that contains a partially double-stranded relaxed circular (rc) DNA. Upon infection, rcDNA is delivered to the nucleus where it is repaired to covalently closed circular (ccc) DNA that serves as the transcription template for all viral RNAs. Our understanding of HBV particle entry dynamics and host pathways regulating intracellular virus trafficking and cccDNA formation is limited. The discovery of sodium taurocholate co-transporting peptide (NTCP) as the primary receptor allows studies on these early steps in viral life cycle. We employed a synchronised infection protocol to quantify HBV entry kinetics. HBV attachment to cells at 4°C is independent of NTCP, however, subsequent particle uptake is NTCP-dependent and reaches saturation at 12 h post-infection. HBV uptake is clathrin- and dynamin dependent with actin and tubulin playing a role in the first 6 h of infection. Cellular fractionation studies demonstrate HBV DNA in the nucleus within 6 h of infection and cccDNA was first detected at 24 h post-infection. Our studies show the majority (83%) of cell bound particles enter HepG2-NTCP cells, however, only a minority (<1%) of intracellular rcDNA was converted to cccDNA, highlighting this as a rate-limiting in establishing infection in vitro. This knowledge highlights the deficiencies in our in vitro cell culture systems and will inform the design and evaluation of physiologically relevant models that support efficient HBV replication.

## 1 Introduction

Hepatitis B Virus (HBV) infects 257 million individuals worldwide and is a major driver of end-stage liver disease, cirrhosis and hepatocellular carcinoma (HCC). HBV is an enveloped DNA and prototypic member of the *hepadnaviridae* that establishes its genome as an episomal, covalently closed circular DNA (cccDNA) in the nucleus of infected hepatocytes. Current treatments suppress viral replication but are not curative, largely due to the persistence of the cccDNA transcriptional template and failure to mount an effective anti-viral immune response (Maini & Pallett, 2018; Rehermann & Thimme, 2019). In most cases, treatment is life-long and patients may still develop HCC (Grossi, Vigano, Loglio, & Lampertico, 2017), highlighting a clinical need for new curative therapies (Revill et al., 2019). Despite its central role in the HBV life cycle our understanding of the host factors regulating cccDNA genesis and half-life is limited (Lythgoe, Lumley, McKeating, & Matthews, 2020).

Viral entry into a host cell represents the first step in the infectious life cycle and is mediated via specific interactions between virus encoded proteins and cellular receptors that define internalisation pathways (Cossart & Helenius, 2014). The discovery that sodium taurocholate co-transporting polypeptide (NTCP) acts as a receptor for HBV (Ni et al., 2014; Yan et al., 2012) enabled the development of in vitro culture systems that support the complete HBV replication cycle. HBV encodes three envelope glycoproteins, small (S), middle (M) and large (L) (Hayes et al., 2016). The preS1 domain of the L protein binds heparan sulfate proteoglycan (HSPG) (Inoue, Ninomiya, Shimosegawa, & McNiven, 2018; Lempp & Urban, 2014; Schulze, Gripon, & Urban, 2007) that precedes high-affinity virus interaction with NTCP. Synthetic peptides mimicking the preS1 binding site for NTCP, such as Myrcludex-B (MyrB) inhibits HBV infection (Li & Urban, 2016; Watashi, Urban, Li, & Wakita, 2014) and a recent phase II clinical trial efficacy in HBV patients co-infected with hepatitis delta virus (Loglio et al., 2019). To date the role NTCP plays in HBV internalisation is not well defined and the virus has been reported to use both clathrin and caveolin-dependent endocytic pathways (Herrscher et al., 2020; Huang, Chen, Chang, Tao, & Huang, 2012; Macovei et al., 2010; Zhang, Zehnder, Damrau, & Urban, 2016). HBV engagement of NTCP was recently shown to activate Epidermal Growth Factor receptor and down-stream signalling pathway was reported to promote virus translocation to the endosomes via undefined pathways (Iwamoto et al., 2019). Our understanding of the host pathways regulating HBV uptake and intracellular particle trafficking is limited and warrants further investigation.

Current HBV culture systems use high viral inocula (ranging from 500 to 10,000 HBV genome equivalents per cell) and frequently use polyethylene glycol (PEG)-mediated precipitation to initiate infection (Ko et al., 2018; Michailidis et al., 2017; Winer et al., 2017; Yan et al., 2015), suggesting that our in vitro model systems are inefficient and may not recapitulate the liver environment. Asabe et al. (2009) reported that a single HBV particle was sufficient to infect a chimpanzee, illustrating the infectious nature of HBV particles in vivo. To explore the early steps in the HBV life cycle required to infect human hepatocyte derived cells expressing NTCP we established a synchronised infection protocol to quantify virus internalisation and early intracellular trafficking events. Our studies show a relatively efficient process of HBV internalisation and particle trafficking to the nucleus with >80% of cell-surface attached virus entering permissive cells. However, the conversion of newly imported partially double-stranded relaxed circular DNA (rcDNA) to cccDNA was inefficient, uncovering a rate-limiting step in establishing productive infection of current in vitro model systems.

## 2 Results

### 2.1 Quantifying HBV attachment and internalisation

**Figure 1:**
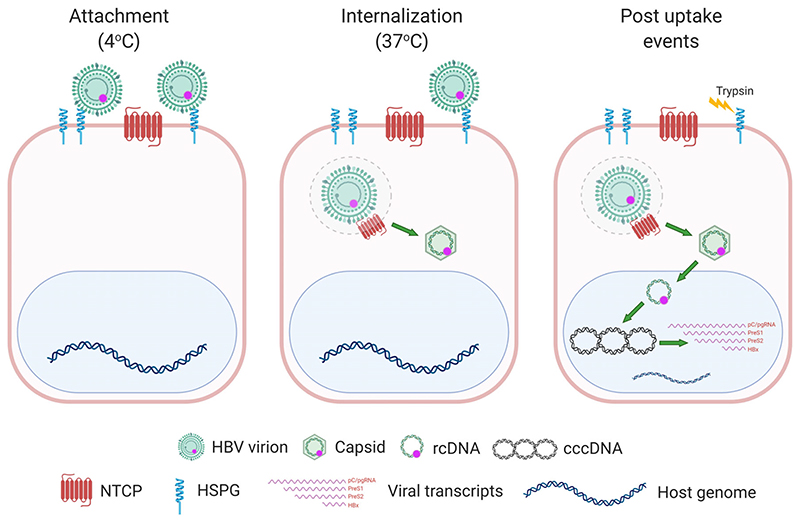
Cartoon depicting a synchronised HBV infection protocol. Pre-chilled cells were inoculated with HBV for 1 h and cells moved to 37°C, leading to synchronous internalisation of viral particles. At various times the cells are treated with trypsin to remove cell-associated non-internalised viral particles and viral parameters quantified

**Figure 2:**
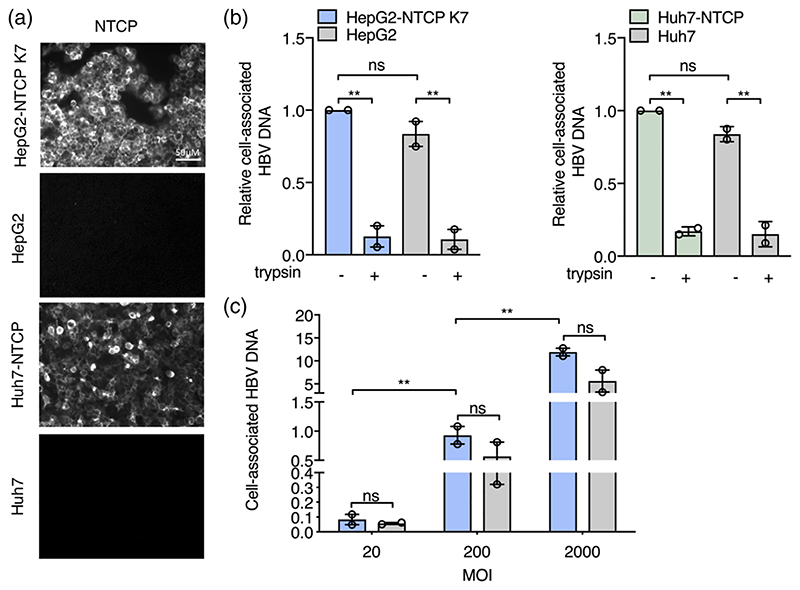
Quantifying HBV attachment to target cells. (a) *HBV attachment to HepG2 cells is NTCP independent*. HepG2-NTCP K7 and Huh7-NTCP along with parental cells were stained for NTCP expression using Atto488 labelled Myrcludex B (200 nM) and imaged using 63× objective (scale bars indicate 20 μm). (b) Pre-chilled HepG2, HepG2-NTCP K7 cells, Huh-7 and Huh7-NTCP were inoculated with HBV (MOI 200) for 1 h on ice, unbound virus removed by washing and cells treated with trypsin or left untreated and cell-associated HBV DNA quantified by RT-PCR. Data is expressed relative untreated HepG2-NTCP cells. (c) *HBV attachment to HepG2 cells is dose-dependent*. Increasing dose of HBV (MOI 20-2000) was inoculated with HepG2-NTCP K7 and HepG2 cells for 1 h at 4°C, unbound virus removed by washing and cell-associated HBV DNA quantified. HBV DNA levels are expressed relative to *PRNP* and represent two independent experiments presented as mean ± standard error of the mean (SEM). Each experiment consisted of three replicates per condition. Statistical analysis was performed using a Mann–Whitney *U* test (*p < .05, **p < .01, ***p < .001)

To quantify HBV attachment and internalisation kinetics we used a well-established method (Funk, Mhamdi, Lin, Will, & Sirma, 2004; Goncalves-Carneiro, McKeating, & Bailey, 2017) where virus is allowed to bind to cells on ice, cultures shifted to 37°C to promote viral uptake and non-internalised virus removed with trypsin (Figure 1). This protocol enables a synchronised uptake of virus particles into target cells that can be enumerated by PCR quantification of HBV rcDNA genomes. HBV was purified on a heparin affinity column (Schulze et al., 2007), followed by sucrose gradient centrifugation (Seitz et al., 2016) and include both mature type-B infectious HBV particles (Seitz et al., 2016) and L-containing filamentous sub-viral particles. This protocol ensures that virus preparations routinely contain less than 1% of non-enveloped naked capsids. Polyethylene glycol 8000 (PEG) is routinely used to enhance HBV infection in cell culture systems including primary human hepatocytes, HepaRG cells and more recently HepG2-NTCP cells (Ko et al., 2018; Michailidis et al., 2017; Winer et al., 2017; Yan et al., 2015). Since this agent precipitates virus and has been reported to promote herpes simplex virus type 1 fusion at the plasma membrane (Walker, Pritchard, Cunha, Aguilar, & Nicola, 2015) and Semliki forest virus (SFV) infection of non-permissive cell types (Arudchandran et al., 1999) all experiments were conducted without PEG. We previously reported that HepG2 cells engineered to express NTCP (Ko et al., 2018) support HBV replication and we selected the K7 sub-clone for our kinetic experiments and confirmed NTCP expression (Figure 2a). Our initial experiments optimised the trypsinization protocol to ensure removal of cell-associated non-internalised virus (Figure 2b). We noted a comparable dose-dependent binding of HBV to HepG2/HepG2-NTCP and confirmed this observation in an independent Huh7 hepatoma line (Figure 2b,c), demonstrating that viral attachment at this low temperature is independent of NTCP.

**Figure 3:**
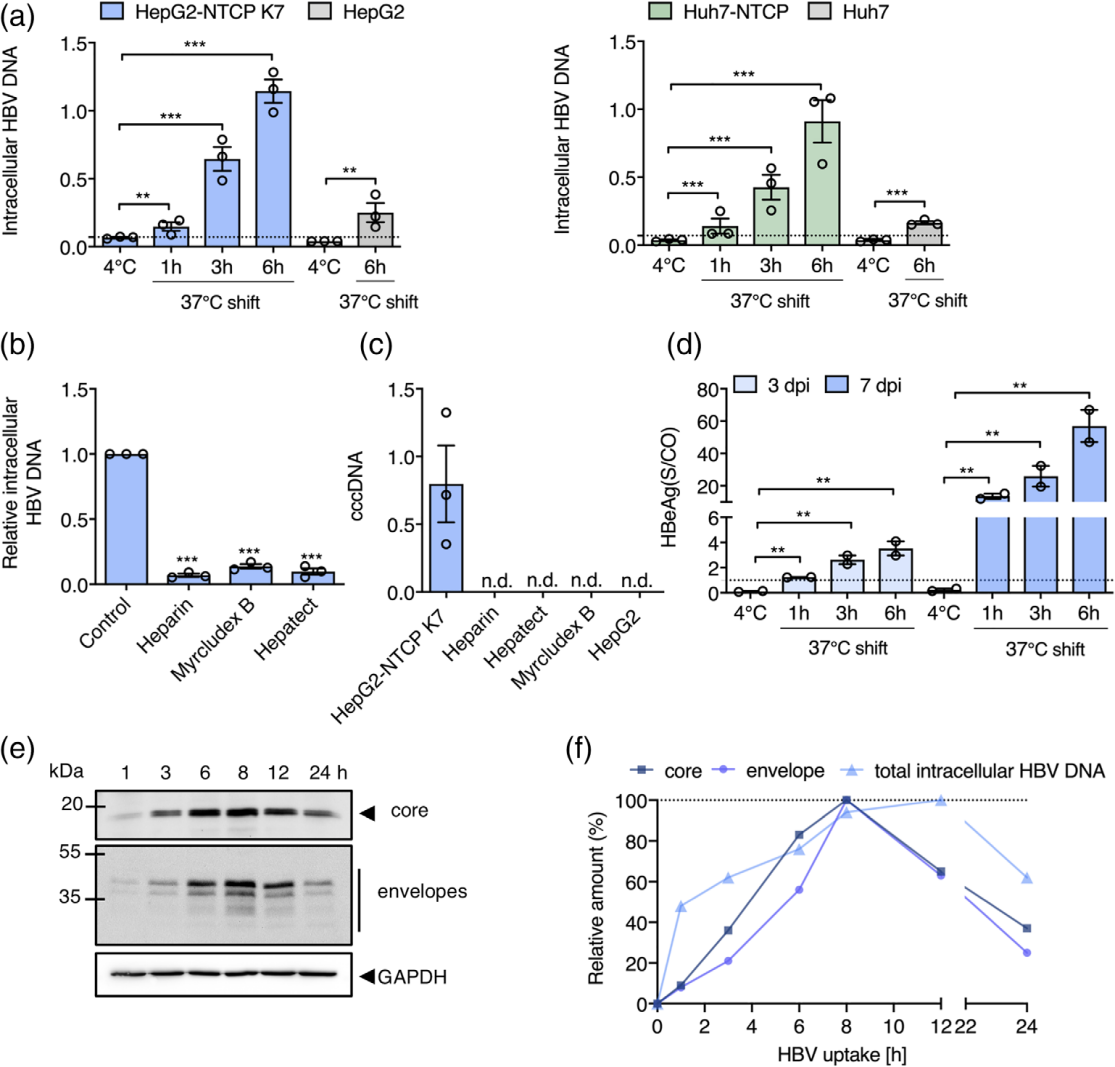
HBV internalisation kinetics. (a) HBV *internalisation is temperature and NTCP-dependent*. HepG2 and Huh-7 hepatoma cells and those engineered to express NTCP were inoculated with HBV (MOI 200) and trypsinized after 1 h at 4°C or following incubation at 37°C for 1, 3 or 6 h. Intracellular HBV DNA levels are expressed relative to *PRNP* and the dotted line represents trypsinized 4°C samples that were set as background for the assay. (b) *Receptor and HBV glycoprotein dependent particle internalisation*. HepG2-NTCP K7 cells were inoculated with HBV (MOI 200) in the presence or absence of heparin (50 IU/mL), Myrcludex B (200 nM) or Hepatect (0.5 IU/mL) and trypsin-resistant intracellular HBV DNA copies measured after 6 h. Data are expressed relative untreated HepG2-NTCP cells. (c) *Short-term synchronised HBV infection of HepG2-NTCP cells generates cccDNA*. Parental HepG2 and HepG2-NTCP K7 cells were inoculated with HBV (MOI 200) as detailed above and after 6 h at 37°C cells were trypsinized and cultured at 37°C for 3 days before measuring cccDNA. Heparin (50 IU/mL), Hepatect (0.5 IU/mL) and Myrcludex B (200 nM) were included as controls. HBV cccDNA levels are expressed relative to *PRNP* and represent three independent experiments presented as mean ± SEM. (d) *Association between internalised HBV particles and HBeAg expression*. HepG2-NTCP K7 were inoculated with HBV (MOI 200) and trypsinized after 1 h at 4°C or following incubation at 37°C for 1, 3 or 6 h and the infected cells cultured for 3 or 7d before measuring extracellular HBeAg. Dotted line represents the limit of detection of the assay, where all values above 1 are considered positive. (e) *HBV internalisation kinetics*. HepG2-NTCP K7 cells were inoculated with HBV (MOI 200) as detailed above (a) and after defined times at 37°C the trypsinized cells were lysed and probed for HBV envelope and core proteins by western blot and images quantified by densitometry. A summary of internalisation kinetics is depicted as the amount of intracellular HBV DNA, core or envelope proteins and plotted as relative data where the highest value of the respective parameter is set to 100%. Data are representative of up to three independent experiments presented as mean ± SEM. Each experiment consisted of three replicates per condition. Statistical analysis was performed using a Mann-Whitney *U* test (*p < .05, **p < .01, ***p < .001), n.d: not detected

To assess the role of NTCP in HBV internalisation we inoculated HepG2 and HepG2-NTCP cells with virus (multiplicity of infection, MOI, of 200 genome equivalents per cell) for 1 h at 4°C and transferred the cultures to 37°C for 1 h, 3 h or 6 h prior to digesting with trypsin to remove non-internalised particles and quantifying intracellular HBV DNA. We observed a time-dependent increase in trypsin-resistant HBV DNA after culturing the cells at 37°C and noted significantly higher levels of viral DNA in HepG2-NTCP after 6 h compared to HepG2 cells (Figure 3a), showing a clear role for NTCP in HBV internalisation. We confirmed these observations with human Huh-7 hepatoma cells that showed similar kinetics and NTCP-dependency of HBV internalisation (Figure 3a). The majority of in vitro studies on HBV replication utilise DMSO to arrest target cell proliferation and we were interested to evaluate the effect of DMSO on HBV internalisation. We noted comparable levels of internalised HBV DNA in DMSO-treated and untreated HepG2-NTCP cells (Supplementary Figure 1), demonstrating a minimal role for DMSO in modulating early steps in viral uptake.

To assess the specificity of our viral internalisation assay we evaluated the effect of known HBV entry inhibitors: heparin that competes for virus attachment to cellular HSPGs (Schulze et al., 2007); MyrB (Schulze, Schieck, Ni, Mier, & Urban, 2010) and Hepatect a polyclonal anti-HBV Ig that neutralises viral infection (Beckebaum et al., 2018). All of the entry inhibitors were used at a concentration that neutralised >90% of HBV infection (assessed by HBeAg expression at 5-day post-infection) and significantly reduced intracellular HBV DNA levels by more than 80% after 6 h post inoculation (Figure 3b). These data confirm that HBV internalisation is dependent on cellular HSPGs, NTCP and viral surface glycoproteins.

Since we noted a low level of HBV internalisation into HepG2 cells we were interested to know if this could establish a productive infection and cultured the cells at 37°C for 3 days and monitored cccDNA levels. We only detected cccDNA in HepG2-NTCP cells, suggesting non-productive uptake pathway(s) in parental HepG2 cells that lack NTCP (Figure 3c). cccDNA formation in HepG2-NTCP cells was blocked by treating cells with Hepatect, heparin or MyrB (Figure 3c). To determine whether early HBV internalisation steps limit productive infection, we cultured HepG2-NTCP cells following 1 h, 3 h or 6 h synchronised infection for 3 and 7 days and measured HBeAg expression as a marker of cccDNA transcriptional activity. It is noteworthy that HBV particles are trypsin sensitive and show an approximate 10-fold reduction in infectivity. We noted a significant association between HBeAg levels and inoculation time that persisted after 3 and 7 days of culture (Figure 3d), demonstrating that the amount of internalised virus defines virus replication.

To extend and validate these observations we assessed HBV internalisation kinetics in HepG2-NTCP cells by monitoring particle associated core or envelope glycoproteins (Figure 3e). Densitometric scanning of western blots showed a peak of intracellular core and surface glycoprotein-associated particles at 8 h and a subsequent decline over the duration of the assay, consistent with our earlier report (Ko et al., 2019). Intracellular HBV DNA showed a delayed peak at 12 h post internalisation. Given the semi-quantitative nature of western blots, these data are in good agreement with earlier PCR data and show a time-dependent internalisation of HBV particles that reaches saturation at 8-12 h post inoculation.

### 2.2 HBV entry kinetics in dHepaRG cells

The bipotent HepaRG cell line can be differentiated towards biliary-like epithelial cells and hepatocyte-like cells that express endogenous NTCP and support HBV replication (Ni & Urban, 2017). In our experience we routinely observe a 1:1 ratio of hepatocyte:biliary cells and staining the differentiated cells for NTCP expression using MyrB showed a low frequency of NTCP expressing cells compared to HepG2-NTCP cells (Supplementary Figure 2A). Attempts to study HBV uptake into dHepaRG cells in the absence of PEG yielded negligible results with no detectable intracellular viral DNA. We previously reported that including PEG 6000 in the inoculum increased HBV uptake 10-fold (Ko et al., 2018) and inoculating dHepaRG cells with HBV in the presence of PEG showed comparable viral uptake kinetics over a 6 h period to HepG2 and Huh-7 cells over-expressing NTCP (Supplementary Figure 2B). These data suggest that NTCP expression levels per se have a negligible impact on the kinetics of HBV internalisation but may regulate the absolute levels of internalised virus. Our attempts to study HBV internalisation into primary human hepatocytes (PHHs) yielded poor quality data with limited evidence of viral uptake even with PEG and showed high donor variability.

### 2.3 Cellular pathways regulating HBV internalisation

**Figure 4:**
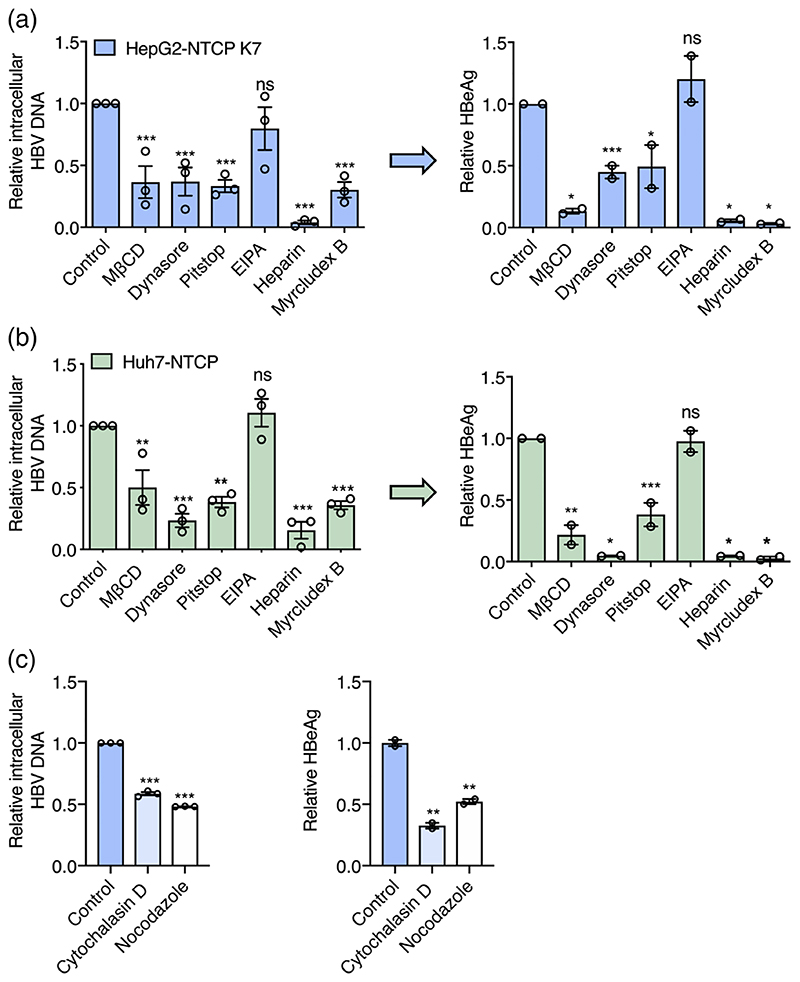
Cellular trafficking pathways exploited by HBV. *HBV internalisation is clathrin and dynamin dependent*. HepG2-NTCP K7 (a) or Huh7-NTCP (b) cells were treated with pharmacological agents targeting dynamin (Dynasore: 100 μM), Clathrin-mediated endocytosis (Pitstop: 50 μM) or macropinocytosis (EIPA: 100 μM) and inoculated with HBV (MOI 200) as detailed in Figure 1. Cells were pre-treated with Dynasore and Pitstop for 0.5 h prior to infection and during the HBV inoculation step. EIPA was co-treated during HBV inoculation. Trypsin-resistant intracellular HBV DNA copies after 6 h or extracellular HBeAg expression after 5 days was measured. Data are plotted relative to untreated control and represent up to three independent experiments presented as mean ± SEM. (c) *HBV internalisation is actin and tubulin dependent*. HepG2-NTCP cells were treated with actin and tubulin disrupting agents, Cytochalasin D and Nocodazole (50 μM each) respectively and inoculated with HBV (MOI 200). Trypsin-resistant intracellular HBV DNA after 6 h or extracellular HBeAg levels after 5 days was measured. Data are plotted relative to untreated control and represent up to three independent experiments presented as mean ± SEM. Each experiment consisted of three replicates per condition. Statistical analysis was performed using a Mann-Whitney *U* test (*p < .05, **p < .01, ***p < .001)

To study the cellular pathways that regulate HBV internalisation we evaluated a panel of pharmacological agents that target various cellular trafficking pathways: MβCD depletes cholesterol from the plasma membrane, Dynasore inhibits dynamin and arrests vesicle formation from the plasma membrane (Macia et al., 2006); Pitstop 2 inhibits clathrin-mediated endocytosis (von Kleist et al., 2011) and EIPA targets the Na^+^/H^+^ exchanger, inhibiting macropinocytosis (Devadas et al., 2014; Dowrick, Kenworthy, McCann, & Warn, 1993). We showed that all agents at the doses previously reported to affect cellular trafficking pathways had a minimal effect on cell viability (Supplementary Figure 3). Vesicular stomatitis virus (VSV) is well recognised to infect cells via clathrin-dependent endocytic pathways (Sun, Yau, Briggs, & Whittaker, 2005) and we used lentiviral pseudoparticles expressing VSV G glycoprotein (VSVpp) to confirm the specificity of these drugs. This demonstrated that MβCD, Pitstop and Dynasore inhibit VSVpp infection of HepG2-NTCP cells (Supplementary Figure 4). All agents were evaluated for their ability to inhibit HBV internalisation into HepG2-NTCP or Huh-7 NTCP cells after 6 h inoculation and HBeAg expression measured after 5 days. Heparin and MyrB were included as positive controls known to inhibit HBV uptake. MβCD significantly reduced HBV uptake and infection, suggesting a role for cholesterol in the HBV internalisation pathway. Pre-treating cells with Dynasore and Pitstop and maintaining compounds during the viral inoculation stage inhibited HBV uptake and infection of both cell lines (Figure 4a,b), demonstrating a dynamin and clathrin dependent endocytic uptake process. In contrast, EIPA, had no effect on HBV internalisation, suggesting a negligible role for macropinocytosis in viral uptake into hepatoma cells. To evaluate whether these agents only affected the HBV uptake process and not viral replication, we added the various drugs after a 1 h internalisation step and showed no change in HBeAg at 7 days postinfection, consistent with these drugs affecting viral uptake into cells (Supplementary Figure 5).

To study the role of the host cytoskeleton in regulating HBV uptake, HepG2-NTCP cells were treated with either Nocodazole or Cytochalasin D that interfere with microtubule and actin dynamics, respectively (Cooper, 1987; Wilson, Panda, & Jordan, 1999) (Figure 4c). Both of these agents had no impact on cell viability (Supplementary Figure 3) and significantly reduced HBV uptake in the first 6 h following infection and HBeAg levels, suggesting a role for microtubule and actin filaments in regulating intracellular capsid trafficking.

### 2.4 Kinetics of HBV trafficking from the cytoplasm to the nucleus

**Figure 5:**
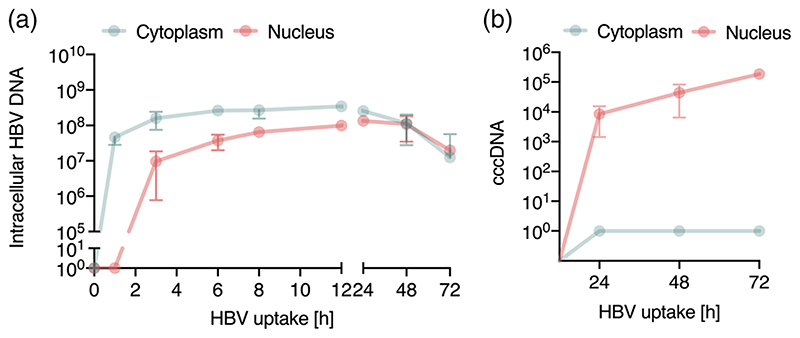
Kinetics of HBV trafficking to the nucleus. (a) *Intracellular HBV trafficking in cytoplasm and nucleus*. Synchronised HBV infection (MOI 200) where cytoplasmic and nuclear samples were harvested at the indicated times. HBV DNA was first detected in cytoplasm at 1 h and in the nucleus after 3 h and monitored thereafter. Detection threshold of the qPCR lies between 10-100 copies. (b) *Synchronised infection and cccDNA levels*. HepG2-NTCP K7 cells were inoculated with HBV (MOI 200) as detailed above (a) and cccDNA levels in the cytoplasmic and nuclear fraction measured. Absolute numbers of total intracellular HBV DNA and cccDNA in cells/cm^2^ are shown. Data are representative of two independent experiments presented as mean ± SEM. Each experiment consisted of duplicates per condition. Statistical analysis was performed using a Mann-Whitney *U* test (*p < .05, **p < .01, ***p < .001)

To study the early steps in the HBV life cycle that precede cccDNA genesis we analysed subcellular fractions for intracellular HBV DNA. A synchronised infection was performed and cytoplasmic and nuclear fractions harvested at early (1, 3, 6, 8 and 12 h) and late (24, 48 and 72 h) time points following trypsinization for quantification of HBV DNA and cccDNA. Cellular fractionation was confirmed by probing cytoplasmic and nuclear samples for α-tubulin and lamin A/C (Supplementary Figure 6A), respectively and DNA samples for presence of the housekeeping gene *PRNP* (Supplementary Figure 6B). Intracellular HBV DNA was first detected in the cytoplasm within 1 h of incubating the cells at 37°C and particle trafficking to the nucleus was detected after 3 h (Figure 5a). HBV DNA levels in the nucleus and cytoplasm were saturated by 12 h and we noted 3.5-fold higher levels of viral DNA in the cytoplasm compared to the nucleus as well as a loss of viral DNA in both nuclear and cytoplasmic fractions after 24 h (Figure 5a). We used published PCR methodologies (Ko et al., 2018) to quantify cccDNA in the nuclear and cytoplasmic fractions and first detected cccDNA in the nuclear fraction 24 h post-infection, which subsequently increased throughout the duration of the experiment (Figure 5b).

### 2.5 Identifying rate-limiting steps in HBV infection

Having optimised the synchronised infection protocol, we quantified HBV attachment (1 h at 4°C), internalisation (6 h, the inferred half-maximal value) and cccDNA (72 h) levels in HepG2 and HepG2-NTCP cells. Similar levels of virus inocula attached to HepG2-NTCP and HepG2 at 4°C (25% and 17%, respectively), consistent with a role for HSPGs in defining the initial association of virus with the cell surface (Table 1). The majority (84%) of cell-bound particles entered HepG2-NTCP cells. We observed a surprisingly high level (46%) of intracellular HBV DNA in HepG2 cells and since we failed to detect any cccDNA in these cells, this most likely reflects a non-productive uptake pathway (Table 1). Finally, we noted that less than 1% of the intracellular HBV DNA detected at 6 h was converted into cccDNA by 72 h. It is worth noting that the detection limit of our assays for quantifying rc- and cccDNA was 100 copies per reaction, suggesting that our earlier conclusion was not biased by differences in the sensitivity of the PCR methods. In summary, these data show that particle internalisation is efficient with the majority of cell-bound particles entering NTCP expressing cells, with at least 22% of particle-associated DNA reaching the nucleus within 12 h. In contrast, the subsequent conversion of incoming rcDNA to cccDNA is inefficient, identifying a rate-limiting step in establishing productive infection.

## 3 Discussion

Our current knowledge on the early steps of HBV infection is not well defined, despite their key role in determining tissue and species tropism. To address this gap we developed an assay to quantify particle internalisation and nuclear transport to identify rate-limiting steps in the early viral life cycle. HBV showed comparable binding to prechilled HepG2 and Huh7 cells independent of NTCP expression, consistent with a role for HSPG in mediating low affinity attachment of HBV to target cells (Schulze et al., 2007; Sureau & Salisse, 2013). A recent report using recombinant HBV particles confirmed the HSPG-dependency of particle attachment and suggested an intracellular role for NTCP (Somiya et al., 2016).

Our kinetic studies show a clear role for NTCP in regulating HBV uptake into HepG2 and Huh-7 cells and for establishing a productive infection. We noted a time dependent increase in particle uptake quantified by measuring intracellular HBV DNA or virus-associated core and envelope proteins that saturated after 12 h. The time for viral DNA and proteins to reach their maximum levels may reflect differential half-lives of the genomic material and protein. HBV DNA was first detected in the cytoplasm after 1 h and in the nuclear fraction by 3 h. HBV core protein encodes a nuclear localisation sequence that targets capsids to the nuclear pore complex (NPC) in an importin α/β mediated manner (Gallucci & Kann, 2017; Kann, Sodeik, Vlachou, Gerlich, & Helenius, 1999; Rabe, Vlachou, Pante, Helenius, & Kann, 2003), however, these studies did not address the time for HBV capsids to reach the NPC. Similar entry kinetics were reported for duck hepatitis B infection (DHBV) of primary duck hepatocytes, showing DHBV DNA in the nucleus by 4 h (Qiao, Scougall, Duszynski, & Burrell, 1999). Importantly, these data are consistent with reports of intracellular trafficking times for other enveloped viruses including human immunodeficiency virus (HIV) that can target the nucleus within 4 h of infection (McDonald et al., 2002; Meyerhans, Breinig, Vartanian, & Wain-Hobson, 2003).

Recent advances in imaging technologies can visualise the internalisation of virus particles (reviewed in [Wang, Burckhardt, Yakimovich, & Greber, 2018]). Several studies have reported the use of fluorescent labelled HBV viral structural proteins or sub-viral particles (Hao et al., 2011; Zhang et al., 2016). However, these approaches come with certain constraints as the labelling can impair viral infectivity and do not always resemble natural infection. Our attempts to image HBV large (L) envelope protein of internalised virus provided weak signals within first 3 h of infection. Combinatorial approaches to visualise HBV envelope and genome may provide a better understanding of the kinetics and location of viral fusion, uncoating and nuclear transport. Herrscher et al. recently reported HBV particles in clathrin-coated pits and vesicles using electron microscopy (EM) and cryo-EM with immunogold labelling (Herrscher et al., 2020) consistent with a clathrin-dependent endocytic entry route.

We first detected cccDNA in the synchronised infection assay after 24 h, consistent with reports for DHBV infection (Qiao et al., 1999). Since our PCR method to quantify cccDNA uses a T5 exonuclease to remove non-cccDNA species, this treatment may result in a loss of >20% of cccDNA (Ko et al., 2018). Given these caveats our data suggest that a minority of intracellular encapsidated rcDNA (<1%) is converted to cccDNA. The slow conversion of rcDNA to cccDNA may simply reflect the rate of genome uncoating and trafficking across the nuclear membrane. However, our fractionation studies demonstrated that up to 22% of total intracellular DNA is in the nucleus within the first 6-12 h of infection suggesting that nuclear targeting is not rate limiting for cccDNA genesis. The mechanism of rcDNA conversion to cccDNA is not fully defined and a number of host pathways have been reported (Gao & Hu, 2007; Hu, Protzer, & Siddiqui, 2019; Mitra, Thapa, Guo, & Block, 2018; Wei & Ploss, 2020). The viral polymerase is removed by Tdp2 (Cui et al., 2015; Koniger et al., 2014; Ni et al., 2014) and this is followed by the removal of the RNA primer by a cellular flap-like structure specific endonuclease, Fen1 (Hu et al., 2019; Kitamura et al., 2018).

A recent study identified five core components of lagging-strand synthesis that were essential for cccDNA formation: proliferating cell nuclear antigen (PCNA), the replication factor C (RFC) complex, DNA polymerase δ, flap endonuclease 1 and DNA ligase 1 (Wei & Ploss, 2020). We were interested to know whether these genes show variable expression in HepG2 or Huh7 compared to other human hepatoma lines and a comparative RNA-sequencing approach enabled us to compare transcript levels across these cell lines (Supplementary methods and Supplementary Figure 7). Normalising read counts to internal housekeeping genes allowed comparison across the cell lines and showed relatively homogenous gene expression (Supplementary Figure 7). PCNA was the most highly expressed gene in all cell lines, with the exception of SNU182. Some factors, such as POLD3, POLD4 and POLK were almost undetectable in HepG2 cells and could limit HBV replication. Immunodeficient mice grafted with human hepatocytes support robust HBV replication (Allweiss & Dandri, 2016; Bissig et al., 2010; Thomas & Liang, 2016) and our recent RNA-sequencing of chimeric human liver mice (Liu et al., 2020) provide an ideal dataset to compare human hepatocyte gene transcript levels with the hepatoma lines. Human hepatocytes from transplanted mice showed a different gene expression profile compared to the hepatoma lines and expressed higher POLD2 and RFC1 transcripts (Supplementary Figure 7). Since many of the DNA repair pathways are ubiquitously expressed these preliminary studies highlight the need for further research to study the role of the DNA repair pathway in rcDNA-cccDNA conversion using recently reported cell-free assays (Wei & Ploss, 2020).

Our results support a role for a clathrin and dynamin in defining HBV particle uptake into HepG2-NTCP and Huh-7-NTCP cells, consistent with a recent report assessing these pathways in HBV infection (Herrscher et al., 2020). In contrast, EIPA had no detectable effect on HBV uptake, suggesting a minimal role for macropinocytosis. This contrasts to observations reported by Macovei et al. (2010) showing a role for caveolin-1 in HBV infection of dHepaRG cells. These differences may be the result of infection protocols, where PEG-enhanced infection may promote viral aggregation and non-physiological uptake pathways. In addition, we and others (Herrscher et al., 2020) noted low to undetectable levels of caveolin-1 expression in HepG2-NTCP cells and PHHs compared to dHepaRG cells, which may also contribute to the different entry pathways reported in these studies. In contrast to many other enveloped viruses that enter cells via clathrin mediated endocytosis, HBV uptake kinetics is slower, for example VSV and SFV require only several minutes to enter their target cell and establish infection (Helenius, Kartenbeck, Simons, & Fries, 1980; Johannsdottir, Mancini, Kartenbeck, Amato, & Helenius, 2009). MβCD reduced HBV entry and infection, suggesting a requirement for cholesterol in the early steps of the HBV life cycle. In line with this finding we previously reported that cholesterol was required for viral infection (Bremer, Bung, Kott, Hardt, & Glebe, 2009). Inhibitors like ezetimibe that interfere with the hepatic cholesterol uptake inhibit HBV infection (Lucifora, Esser, & Protzer, 2013), supporting a role for cholesterol in HBV infection.

In summary, our studies show the majority of cell bound particles enter NTCP expressing target cells, however, only a minority of intracellular rcDNA is converted to cccDNA, highlighting this as a rate-limiting step in establishing infection in vitro. We believe this knowledge is essential to aid the interpretation of mechanistic studies probing pathways regulating cccDNA genesis and half-life and for screening anti-viral agents. Furthermore, this data will inform the design of physiologically relevant models that support efficient HBV replication.

## 4 Experimental Procedures

### 4.1 Cell lines

HepG2-NTCP K7 cells and Huh-7 NTCP cells (Ko et al., 2018; Meredith et al., 2016) were cultured in Dulbecco’s Modified Eagles Medium F12 supplemented with 10% foetal bovine serum (FBS) and penicillin/streptomycin. HepaRG cells were cultured in Williams E medium supplemented with 10% FBS, 50 U penicillin/streptomycin/mL, 5 μg human insulin/mL and 5 × 10^−7^ M hydrocortisone hemisuccinate (Sigma). Cells were seeded and expanded for 2 weeks and differentiated for another 2 weeks in the presence of 1.8% dimethyl sulfoxide (DMSO) (Gripon et al., 2002).

### 4.2 HBV purification protocol

HBV was purified and concentrated from HepAD38 cell culture supernatant using previously published protocols (Burwitz et al., 2017; Seitz et al., 2016). In brief, cells were cultivated in multi-layer flasks and viral particles purified from the supernatant by passing over Heparin HiTrap columns (5 mL) and naked capsids were found in the column flow-through (Schulze et al., 2007). Bound virus was eluted with NaCl (390 mM) and purified by sucrose gradient centrifugation (3 mL 60%, 7 mL 25% and 9 mL 15%) at 32,000 rpm in a SW32Ti rotor. The resulting gradient was fractionated in 2 mL aliquots and infectivity of the virus-rich fraction evaluated in HepG2-NTCP cells and aliquots stored at –80°C (Seitz et al., 2016). Caesium chloride ultracentrifugation can separate naked capsids and fully formed virions and qPCR analysis of fractions for HBV DNA showed that the ratio of naked capsids in the purified viral stocks was less than 1%.

### 4.3 Synchronised HBV infection

Cells were seeded at 1.2 × 10^6^ or 3 × 10^5^/well, for 6- and 24 well plates, respectively, on collagen-coated plates and differentiated for 2 days with media containing 2.5% DMSO. Cells were pre-chilled on ice for 15 minutes and cold HBV containing inoculum added to cells on ice for 1 h enabling the virus to bind to the cell surface. Medium was exchanged and cells shifted to 37°C for 1-72 h. For harvesting, the cells were washed with PBS and trypsinized for 3 minutes. Samples were either subjected to DNA extraction for HBV DNA or cccDNA analysis or samples collected for HBeAg measurement.

### 4.4 Characterising pathway inhibitors

Cells were seeded at 3 × 10^5^ cells/well and differentiated with 2.5% DMSO for 2 days. Cells were pre-treated with MβCD (10 mM for 2 h); ethyl-isopropyl amiloride (EIPA) (100 μM for 30 min); Dynasore (100 μM for 30 min); Pitstop2 (50 μM for 30 min); Cytochalasin D (50 μM for 2 h) and Nocodazole (50 μM for 2 h) prior to inoculating with HBV in the continued presence of the inhibitors. Total intracellular HBV DNA was measured at 6 h and secreted HBeAg at 3 days post-infection. All inhibitors were purchased from Sigma-Aldrich. Cell viability was analysed using CellTiter-Blue (Promega) according to the manufactures protocol.

### 4.5 VSVpp infection assay

VSVpp were produced as previously reported (Meredith et al., 2016). HepG2-NTCP and Huh7-NTCP cells were seeded in 96-well plates and differentiated with 2.5% DMSO for 2 days. Cells were either pretreated with MβCD (10 mM) and EIPA (100 μM) for 2 h and 0.5 h, respectively, or pre-treated with Dynasore (100 μM) and Pitstop2 (50 μM) for 0.5 h and kept for simultaneous treatment with the VSVpp inoculum. After 24 h cells were washed and luciferase activity measured at 3 days post-infection.

### 4.6 Quantification of HBV DNA and cccDNA by real time PCR

Total cellular DNA was extracted using a NucleoSpin Tissue kit (Macherey Nagel) according to the manufacturers protocol. Intracellular HBV DNA and cccDNA was analysed as previously described in Ko et al. (2018). For cccDNA analysis total DNA was subjected to T5 exonuclease digestion. Total intracellular HBV DNA (HBV1844For: 5’-GTTGCCCGTTTGTCCTCTAATTC-3’ and HBV1745Rev: 5’-GGAGGGATACATAG-AGGTTCCTTGA-3’) and cccDNA (cccDNA92For: 5’-GCCTATTGATTGGAAAGTATGT-3’ and cccDNA2251Rev: 5’-AGCTGAGGCGGTATCTA-3^’^) were normalised to human prion protein *PRNP* (PRNPFor: 5’-TGCTGGGAAGTGCCATGAG-3’ and PRNPRev: 5’-CGG TGCATGTTTTCACGATAGTA-3^’^). An external plasmid standard was used for absolute quantifications.

### 4.7 HBeAg ELISA

Cells were seeded at 3 × 10^5^ cells/well in 24 well plates and differentiated for 2 days with media containing 2.5% DMSO prior to infecting with HBV Supernatant was collected at 3 days post-infection unless stated otherwise. HBeAg was qualitatively quantified using an automated BEP III system (Siemens Healthcare).

### 4.8 Staining for cell-surface expressed NTCP

Cells were seeded 3 × 10^5^ cells/well onto collagen-coated coverslips in 24 well plates. Cells were incubated with Atto488-labelled MyrB (200 nM) for 30 min at 37°C (Lempp et al., 2017; Ni et al., 2014), unbound peptide was removed by washing 2× with PBS and fixed with 4% Paraformaldehyde (PFA) (Roth) subjected to fluorescence microscopy (Olympus FV10i).

### 4.9 Western blotting

Cells were seeded at 1.2 × 10^6^/well in 6-well plates for synchronised HBV infection and cells lysed post-trypsinization at indicated time points with Pierce RIPA buffer (Thermo Scientific Fisher) supplemented with a protease inhibitor cocktail (Hustedt & Durocher, 2016) on ice for 10 min. 4× Laemlli buffer was added to obtain a final 1× concentration and incubated at 95°C for 5 min. Proteins were separated on a 12% polyacrylamide gel and transferred to Polyvinylidene difluoride (PVDF) membranes (Amersham). The membranes were blocked in PBST, 3% milk (Sigma). Rabbit anti-HBcAg (8C9, in house generated) and rabbit anti-HBV envelope protein (H863, provided by H. Schaller), commercially available antibodies specific for anti-lamin A/C (Clone 14, BD Biosciences), GAPDH (Clone 6C5, Acris) and α-tubulin (CloneB-5-1-2,Sigma) were incubated in 1% milk overnight. Proteins were detected using a horse radish peroxidase (HRP) coupled secondary antibody with the Amersham ECL Prime Western Blotting Detection Reagent.

### 4.10 Subcellular fractionation

HepG2-NTCP cells were seeded at 1.2 × 10^6^/well in 6-well plates and harvested for fractionation using NE-PER™ Nuclear and Cytoplasmic Extraction Reagents (Thermo Scientific Fischer) according to manufacturer’s protocol. A synchronised HBV infection was performed and samples collected at the indicated time points post-trypsinization and intracellular DNA isolated from the cytoplasmic or nuclear fractions using NucleoSpin Tissue kit (Macherey Nagel) and HBV DNA and cccDNA quantified by qPCR as described above.

### 4.11 Statistics

All experiments were performed at least twice and replicate numbers provided in figure legends. *p* Values were determined using Mann–Whitney *U*-Test (two group comparisons; unpaired data) using PRISM version 8. In the figures *denotes *p* < .05, ** denotes *p* < .01, ***denotes *p* < .001, ****denotes *p* < .0001.

## Supplementary Material

Supporting Information

## Figures and Tables

**Table 1:** Rate limiting steps in the early HBV life cycle

	HepG2-NTCP K7			HepG2		
	Absolute copy number (per cm^2^)	% From input virus	% From attached virus	Absolute copy number (per cm^2^)	% From input virus	% From attached virus
HBV inoculum	1.5 × 10^8^ ± 2.9 × 10^7^	−	−	1.5 × 10^8^ ± 2.9 × 10^7^	−	−
Attachment	3.7 × 10^7^ ± 4.7 × 10^6^	25 ± 6	−	2.6 × 10^7^ ± 4.1 × 10^6^	17 ± 5	−
Total intracellular HBV DNA (6 h p.i)	3.1 × 10^7^ ± 2.5 × 10^6^	21 ± 6	84 ± 21	1.2 × 10^7^ ± 1.9 × 10^6^	8±3	46 ± 17
cccDNA (72 h p.i.)	2.2 × 10^5^ ± 3.8 × 10^4^	0.15 ± 0.05	0.6 ± 0.16	−	−	−

Note:Summary of synchronised infection in HepG2-NTCP and HepG2 cells. Data showing input HBV DNA of the inoculum, cell-associated HBV DNA (attachment) and intracellular HBV DNA after 6 h post infection (p.i.) at 37°C along with cccDNA levels at 72 h p.i. Absolute copy numbers are presented from cells/cm^2^. These data are presented as the mean ± SEM of eight independent experiment with biological duplicates. n.d: not detected.
