# Supplementary material for: Synchronised infection identifies early rate-limiting steps in the hepatitis B virus life cycle
Source: Cell Microbiol. Author manuscript; Available in PMC 2021 Sep 26. (PMC7611726; doi:10.1111/cmi.13250)
Supplement: Supporting Information [file EMS135042-supplement-Supporting_Information.docx]

**Synchronized infection identifies early rate-limiting steps in the hepatitis B virus life cycle.**

Anindita Chakraborty^1,2^, Chunkyu Ko^1^, Christin Henning^1^, Aaron Lucko^1^,

James M. Harris^3^, Fuwang Chen^1^, Xiaodong Zhuang^3^, Jochen M. Wettengel^1^,

Stephanie Roessler, Ulrike Protzer^1,2,4^ and Jane A McKeating^3*^.

**SUPPLEMENTARY MATERIAL**

**SUPPLEMENTARY EXPERIMENTAL METHODS.**

**Cell lines, RNA extraction and RNA sequencing.**

We investigated 7 hepatocellular cell lines, Hep3B, HepG2, SNU182, HLE, HLF, Huh1, and Huh7. All cell lines were regularly tested to be negative for mycoplasma contamination using MycoAlert (Lonza, Basel, Switzerland) and authenticated by STR analysis. HepG2 and SNU182were cultured in RPMI-1640 medium, HLE, HLF, Huh1, and Huh7 in DMEM medium, and Hep3B in MEM medium. All media were supplemented with 10% fetal bovine serum (Thermo Fisher Scientific, Offenbach, Germany) and 1% Penicillin-Streptomycin (100IU/ml and 100g/ml). RNA was extracted with the NuclosSpin RNA kit (Macherey-Nagel, Düren, Germany) according to the manufacturer’s protocol and integrity assessed using Agilent RNA Nano 6000 chips on the Agilent Bioanalyzer 2100. RNAseq libraries were generated of 1.5 µg total RNA using the NEBNext Ultra II Directional RNA Preparation Kit in conjunction with NEBNext Poly A Selection, and NEBNext Multiplex Oligo (Illumina). Libraries were quantified by Qubit (Thermo Fisher) with the High Sensitivity DNA Assay, and quality checked with the DNA 1000 Chip on the Agilent Bioanalyzer. Samples were equimolar pooled and sequenced on the Illumina HiSeq 2500 in 125 base paired end modus.

**Quality control, pre-processing and statistical analysis of RNA sequencing data.**

FastQC version 0.11.2 (bioinformatics.bbsrc.ac.uk/projects/fastqc) was used to assess the quality of raw reads, which were subsequently filtered using PRINSEQversion 0.20.3 with the quality parameters -min_qual_mean 20 -ns_max_p 10 -trim_qual_right 3 -min_len 30(Schmieder & Edwards, 2011). Variant calling was performed following the GATK guideline for RNAseq data that included a two-pass alignment with STAR (2.5.2b)(Dobin et al., 2013), removal of PCR duplicates with picard tools (2.1.0) (http://broadinstitute.github.io/picard/) and variant calling with GATK 3.5 (McKenna et al., 2010). The GRCh38.90 version of the human genome was used as reference, mapping quality and the coverage distribution were assessed with Qualimap (2.2.1)(Okonechnikov, Conesa, & Garcia-Alcalde, 2016). Gene expression values were quantified as transcripts per million (TPMs) using the output from feature Counts of the Subread package version 1.6.4 (Liao, Smyth, & Shi, 2014).

**
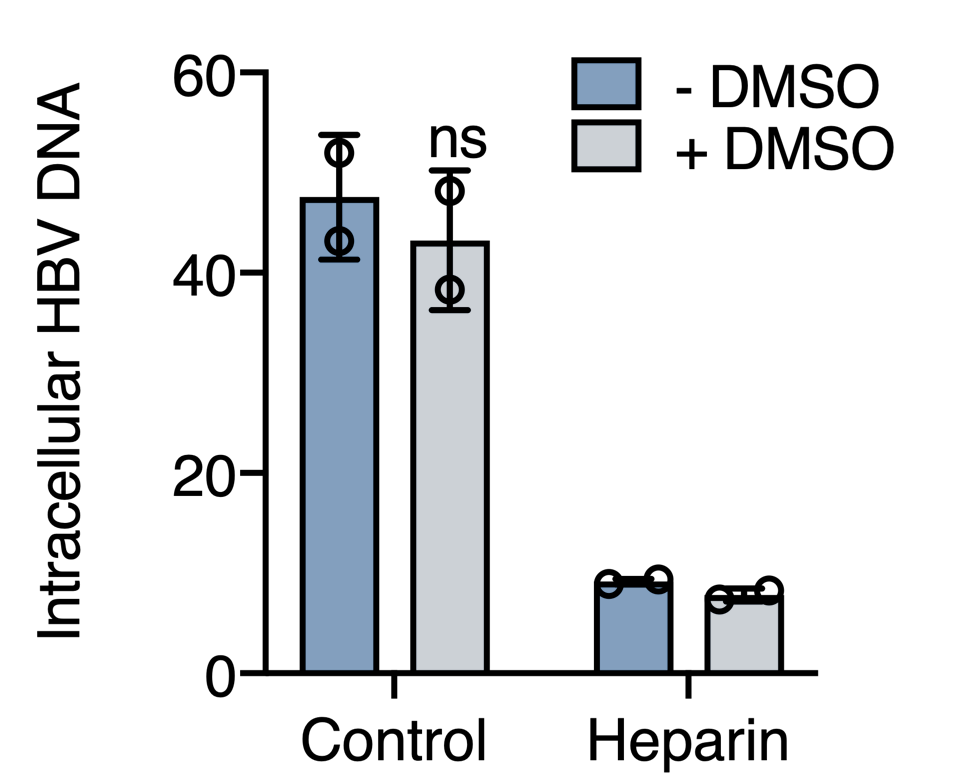
**

**Supplementary Fig.1: Effect of DMSO on HBV entry.** HepG2-NTCP K7 cells were treated with 2.5% DMSO for 3 days (+DMSO) or left untreated (-DMSO) and inoculated with HBV (MOI 200) for 1h on ice and transferred to 37°C for 6h, trypsinized and intracellular HBV DNA quantified. Data is expressed relative to *PRNP*. Heparin (50 IU/ml) was included as control to inhibit HBV uptake. Data is plotted as mean ± SD and derived from 2 independent experiments.


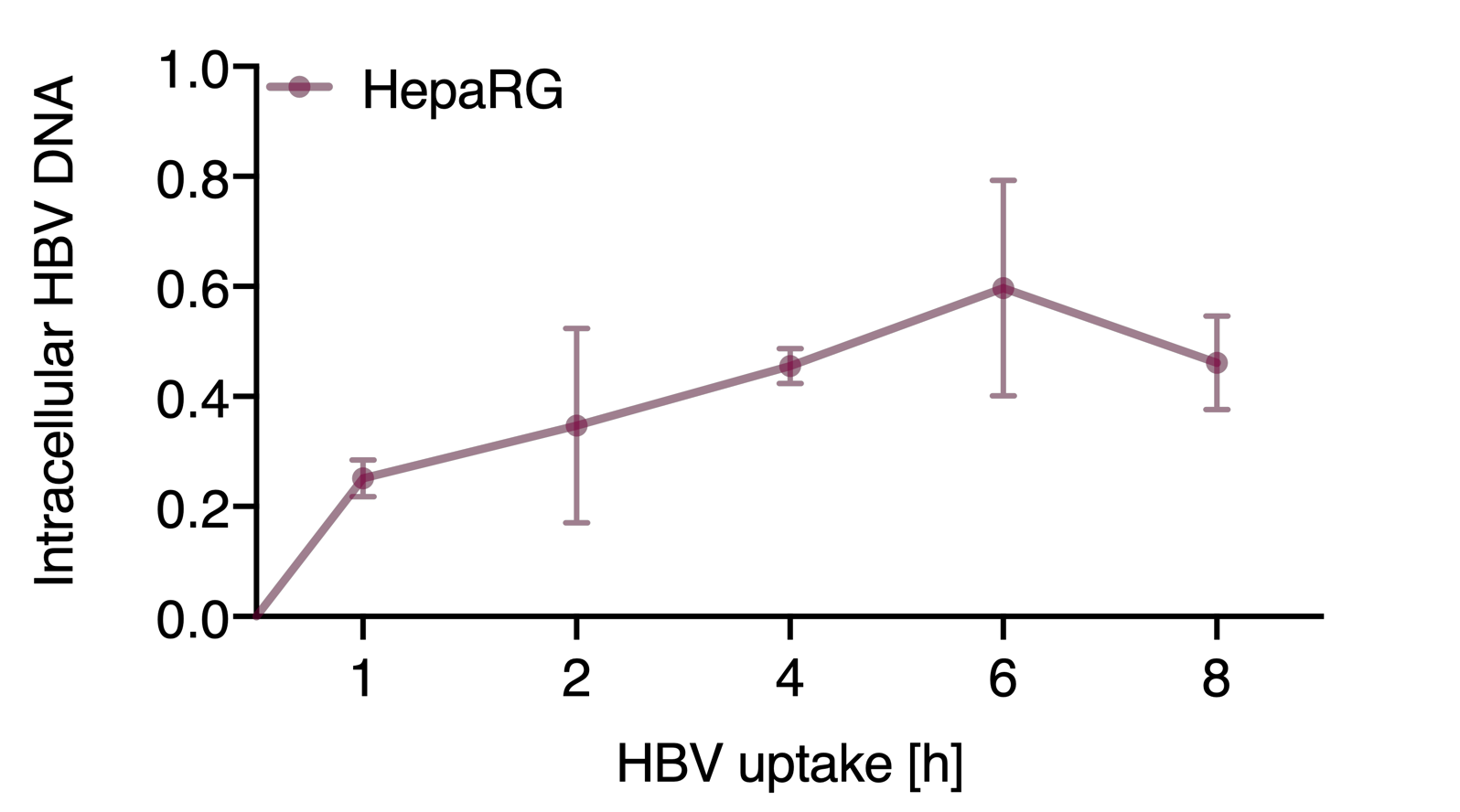

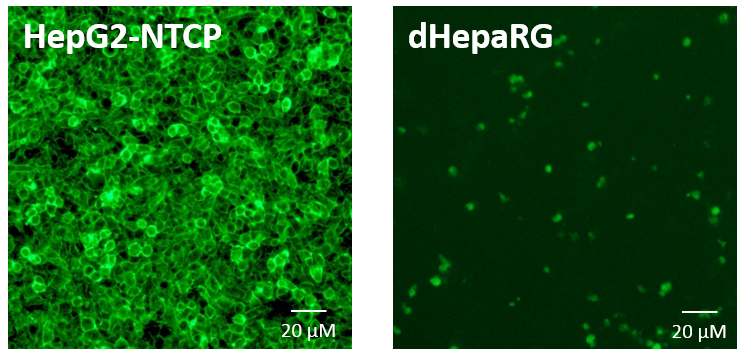


**a**

**b**

**Supplementary Fig.2: HBV internalization into dHepaRG cells.** (**a**) HepG2-NTCP cells and differentiated HepaRG cells (dHepaRG) were stained for NTCP expression with Atto488 labelled Myrcludex B (200 nM) and fluorescent images acquired using a Zeiss fluorescence microscope. (**b**) dHepaRG cells were inoculated with HBV (MOI 200) in the presence of 4% PEG 800 for 1h on ice, unbound virus removed by washing and samples collected at the indicated time points (0-8h) and measured for total intracellular HBV DNA. Data is expressed relative to *PRNP*. Data is plotted as mean ± SD and derived from 2 independent experiments.


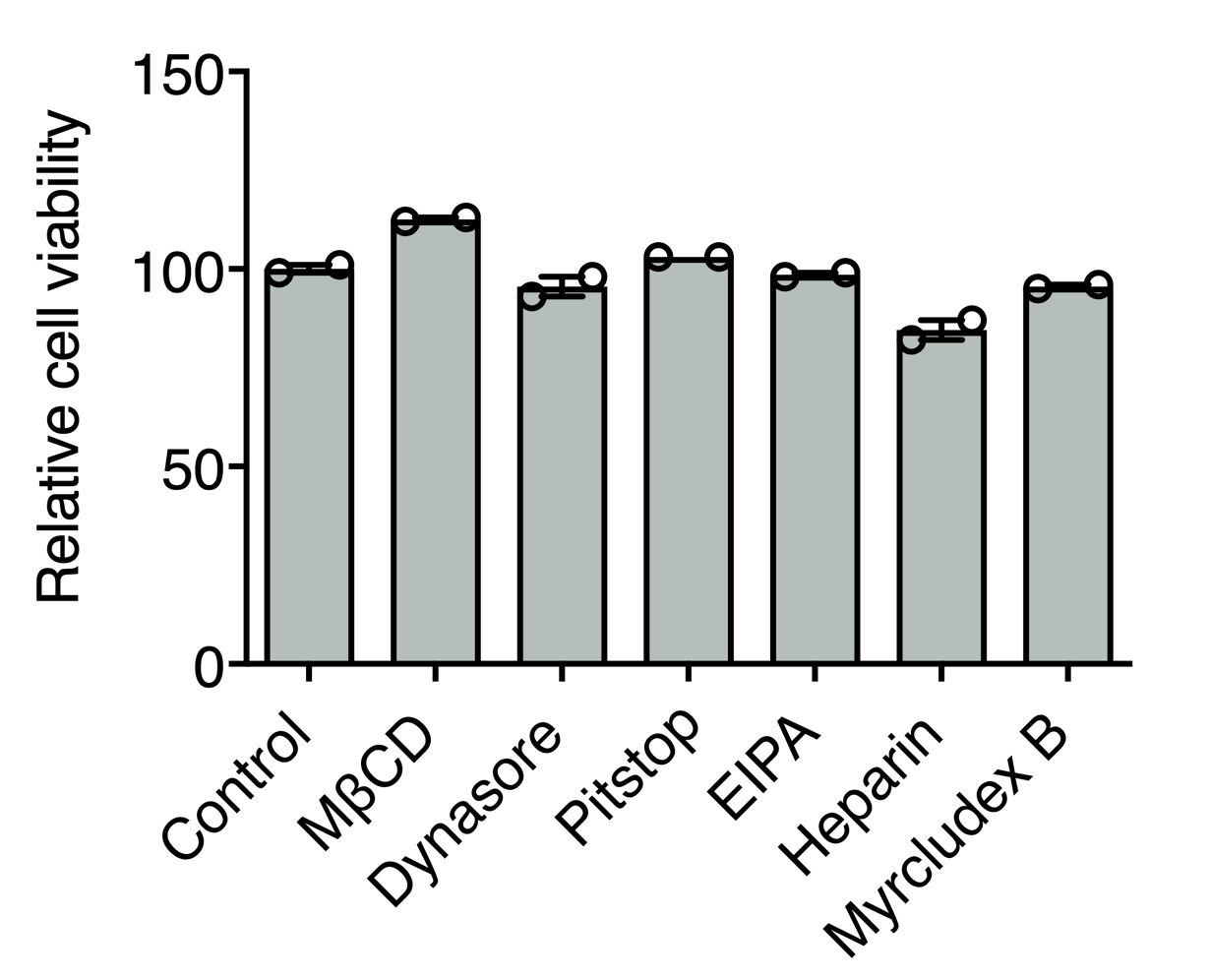

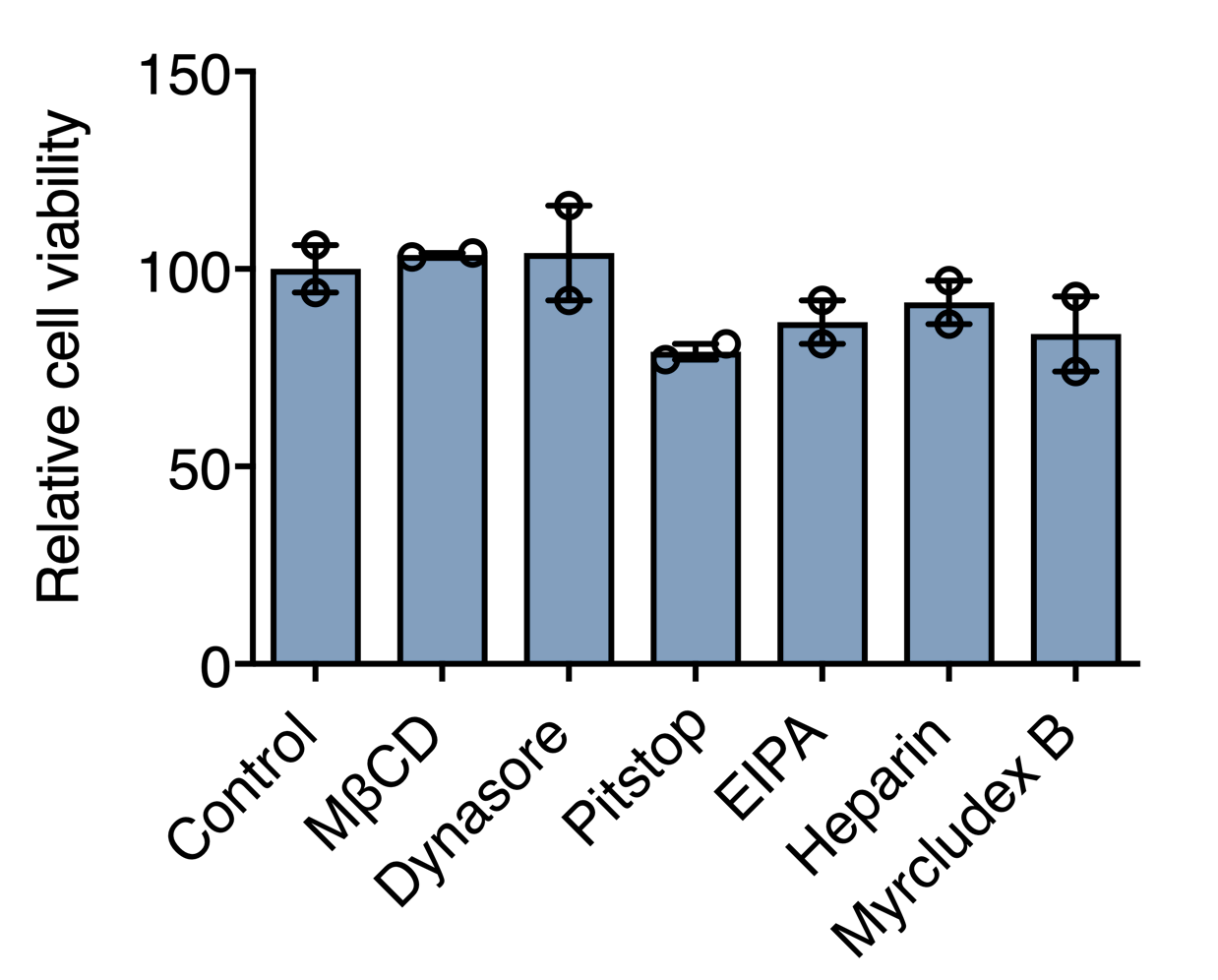

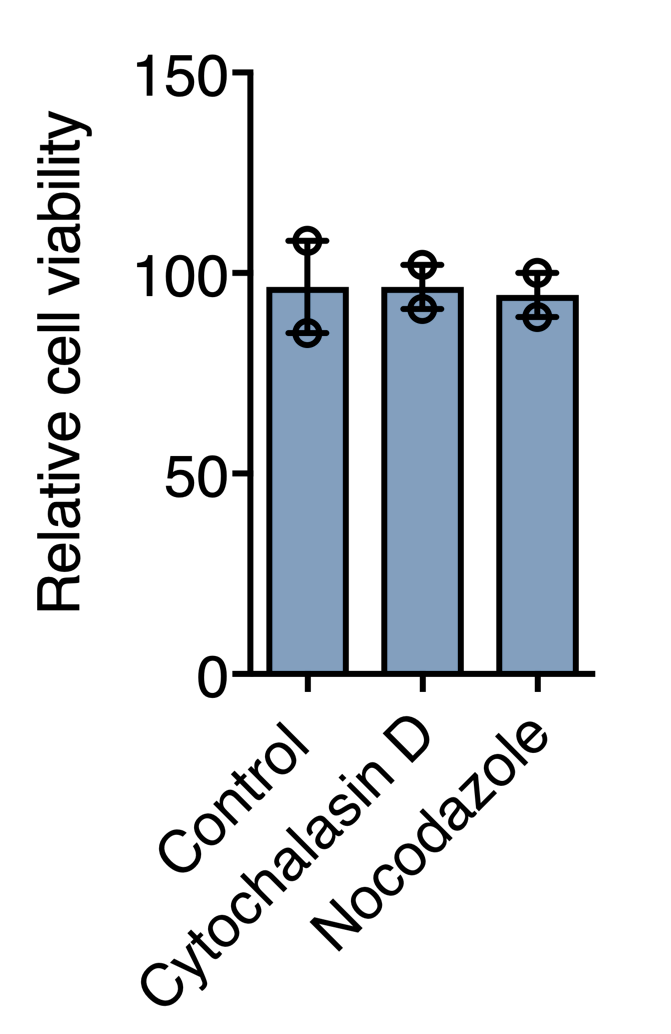


**a**

**b**

**c**

**Supplementary Fig.3: Effect of pharmacological agents on hepatoma cell viability.** Huh7-NTCP (a) and HepG2-NTCP (b-c) were pre-treated with MβCD (10 mM for 2h); Dynasore (100 μM for 30 mins); Pitstop2 (50 μM for 30 mins); EIPA (100 μM for 30 mins); Cytochalasin D (50 μM each) and Nocodazole (50μM each) before inoculating with HBV. After 6h the cells were washed and viability assessed. Data is plotted as mean ± SEM derived from two experiments that consisted of duplicate wells per condition.


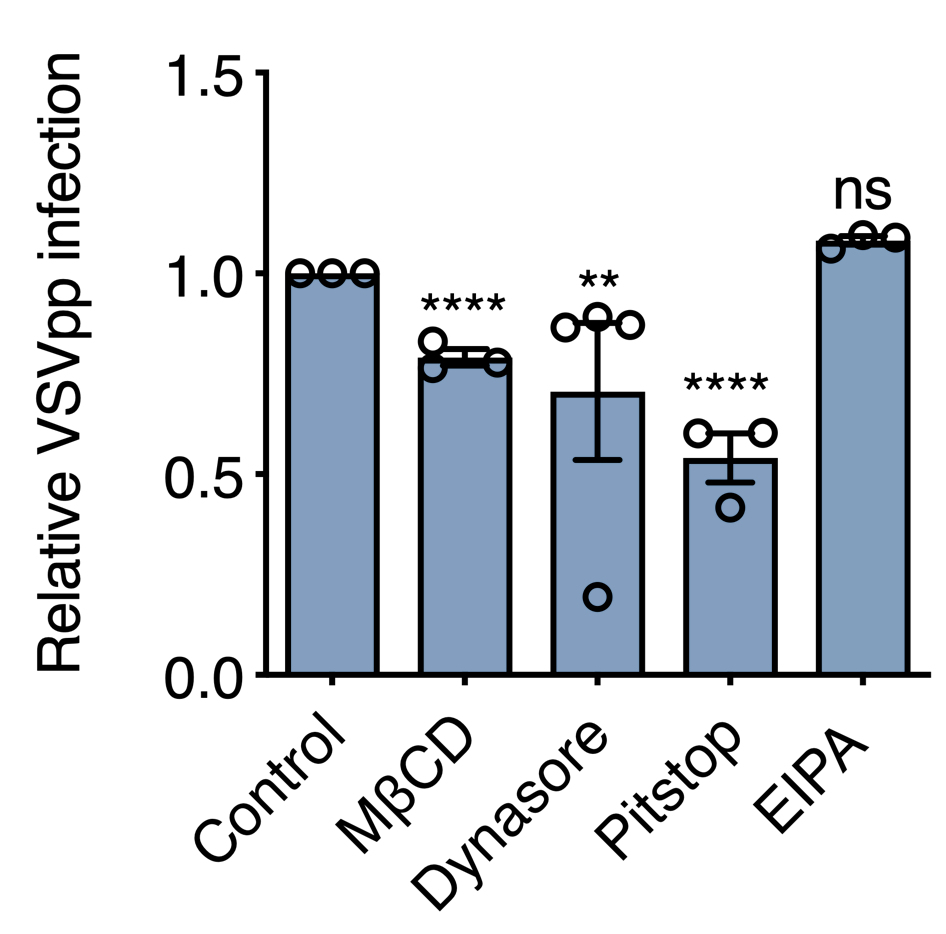


**Supplementary Fig.4: Effect of pharmacological agents on VSV-G pseudoparticle infection**. HepG2- NTCP K7 cells were pre-treated with MβCD (10 mM for 2h); EIPA (100 μM for 30 mins); Dynasore (100 μM for 30 mins) and Pitstop2 (50 μM for 30 mins) before inoculating with VSV-Gpp for 30 mins. The unbound virus was removed by washing and the cultures re-fed with drugs and incubated at 37C for 24h. Cells were lysed and luciferase activity measured. Data is plotted as mean ± SEM derived from three experiments that consisted of quadruplicates per condition. Statistical analysis was performed using a Mann–Whitney U test (t**p<0.01, ***p<0.001, **** p <0.0001: n.s. not significant).


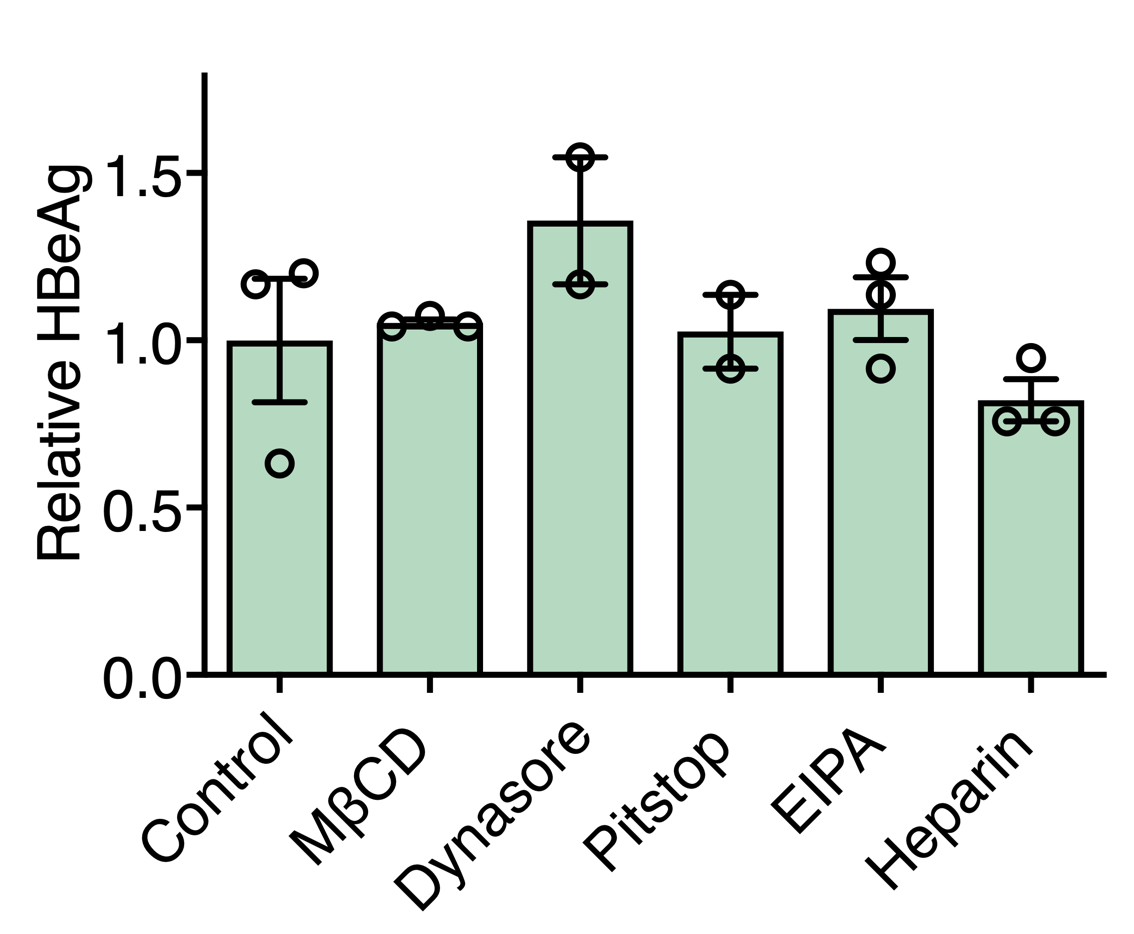

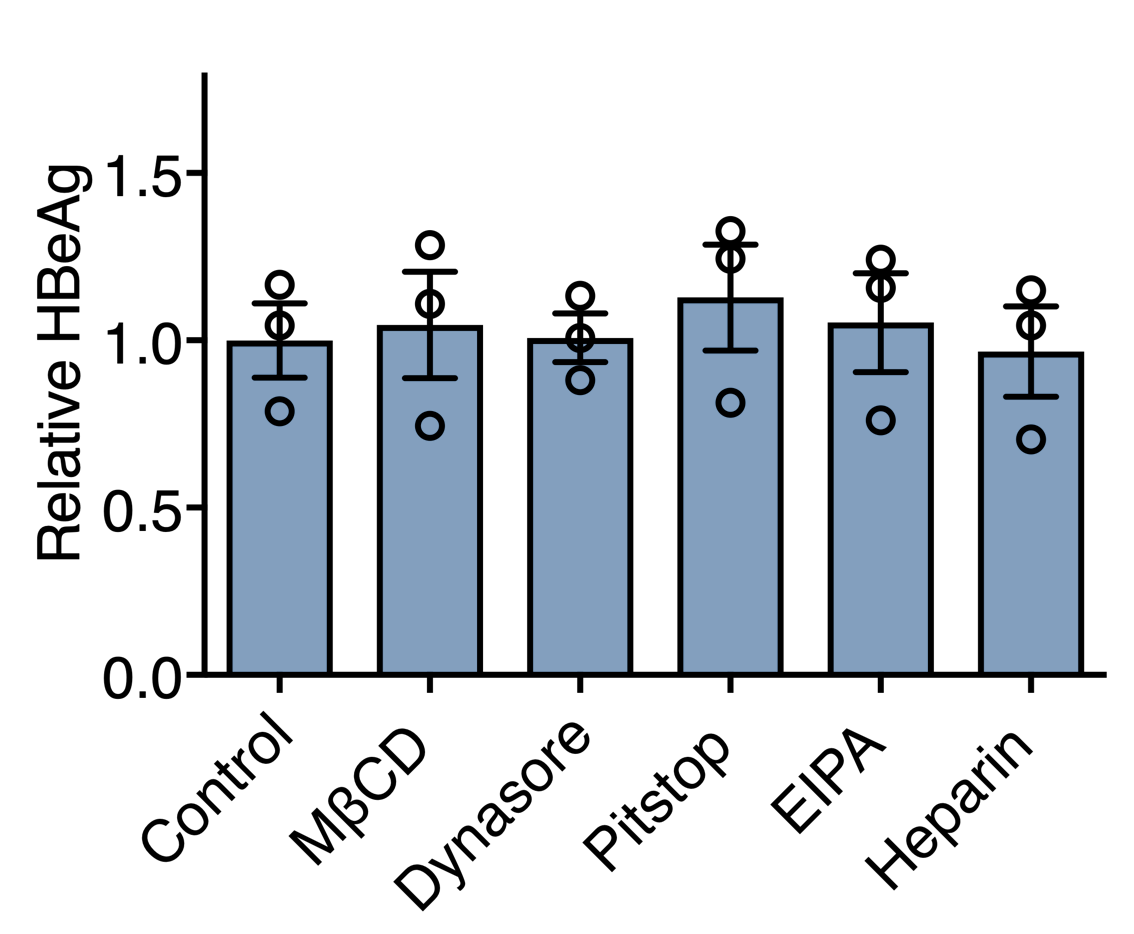


**a**

**b**

**Supplementary Fig.5: Effect of pharmacological agents on HBV replication.** Differentiated HepG2-NTCP K7 (**a**) and Huh7-NTCPcells (**b**) were inoculated with HBV (MOI, 200) for 1h on ice and transferred to 37°C for 1h, trypsinized and media containing MβCD (10 mM), Dynasore (100 μM), Pitstop2 (50 μM) and EIPA (100 μM) added. After 5h the media was replaced with DMSO containing media and the cultures maintained at 37C for 7 days when extracellular media was harvested and HBeAg quantified. Data is plotted as mean ± SEM derived from three experiments. Each experiment consisted of triplicates per condition.


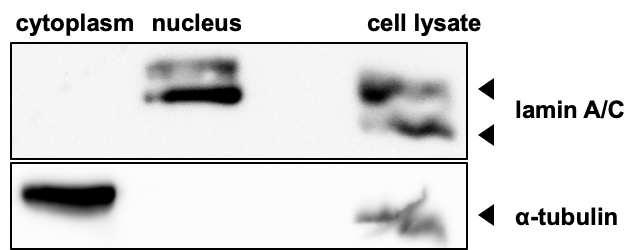

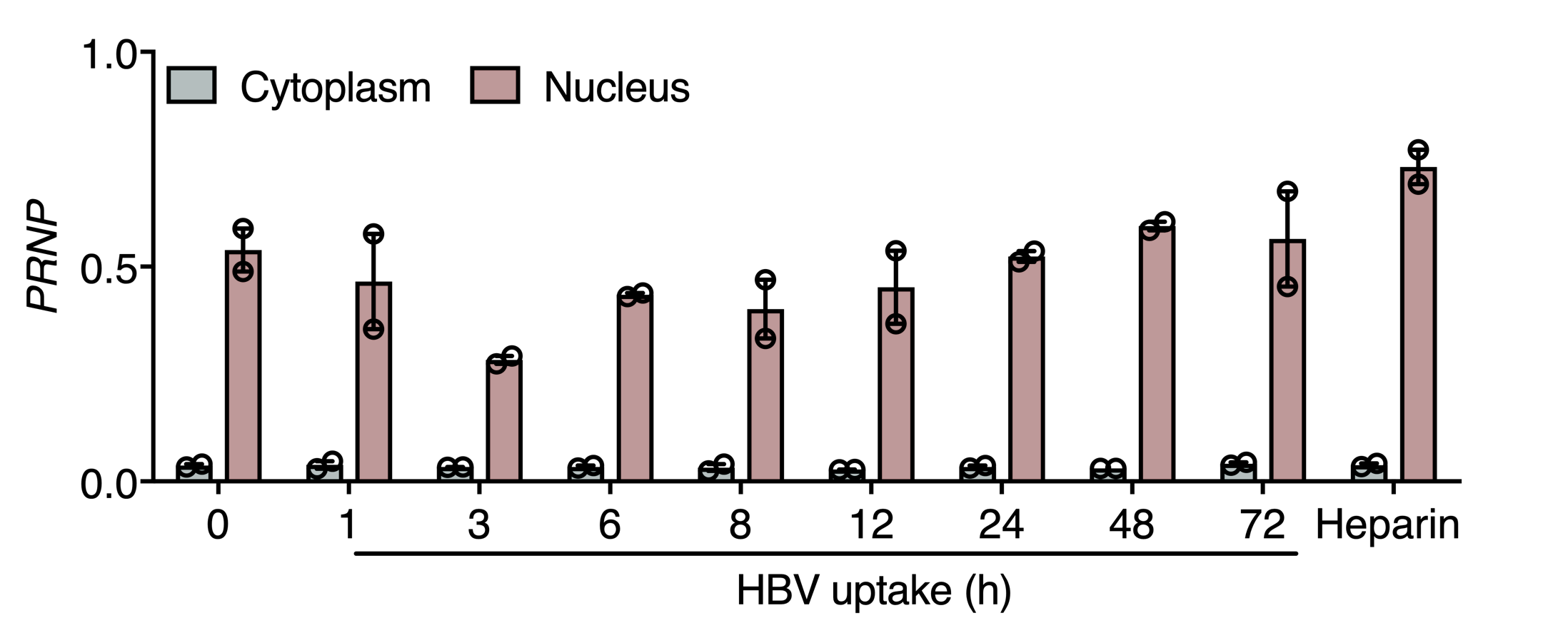


**a**

**b**

**Supplementary Fig.6: Subcellular fractionation.** Samples were collected from a synchronized HBV infection at the indicated time points (0-72h) and cytoplasmic and nuclear fractions collected. Purity of representative fractions was confirmed by western blotting for nuclear and cytoplasmic resident proteins, lamin A/C and a-tubulin, respectively. Whole cell lysate was included as a control (**a**). In addition, DNA was purified from all collected samples for qPCR analysis of housekeeping gene *PRNP* (**b**). Data are representative of two independent experiments presented as mean ± SEM. Each experiment consisted of duplicates per condition.


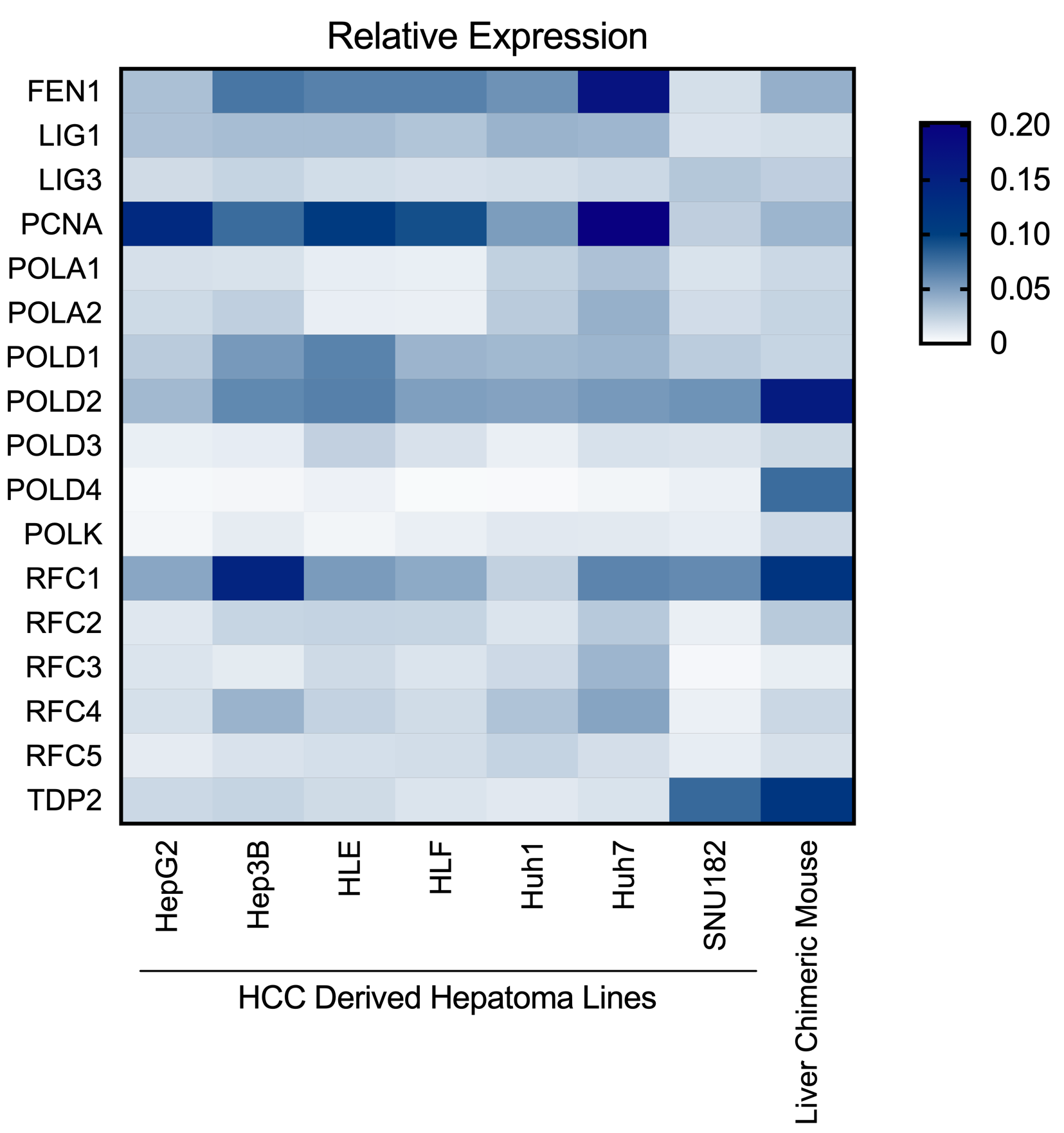


**Supplementary Fig. 7: Transcript levels of essential factors for HBV cccDNA genesis in a panel of human hepatoma lines.** A panel of human hepatoma cell lines were RNA-sequenced and gene expression quantified (doi:10.11588/data/SCGJHQ). Human liver chimeric mouse tissue (n=3) was sequenced as described (https://www.biorxiv.org/content/10.1101/2020.03.27.011841v1). Data were analysed for transcript levels of recently described human genes essential for cccDNA formation (Hu, Protzer, & Siddiqui, 2019; Wei & Ploss, 2020), where gene expression is expressed relative to the geometric mean of 2 house-keeping genes (RPLP0 and  β-Actin). Factors are listed in alphabetical order. *FEN1,* Flap structure-specific endonuclease 1*; LIG1,* DNA ligase 1*; LIG3,* DNA ligase 3*; PCNA,* Proliferating cell nuclear antigen*; POLA1-2,* DNA polymerase alpha 1-2*; POLD1-4,* DNA polymerase delta 1-4*; POLK,* DNA polymerase kappa*; RFC1-5,* Replication *; TDP2,* Tyrosyl-DNA phosphodiesterase 2*.*

**REFERENCES**

Dobin, A., Davis, C. A., Schlesinger, F., Drenkow, J., Zaleski, C., Jha, S., Gingeras, T. R. (2013). STAR: ultrafast universal RNA-seq aligner. *Bioinformatics, 29*(1), 15-21. doi:10.1093/bioinformatics/bts635

Hu, J., Protzer, U., & Siddiqui, A. (2019). Revisiting Hepatitis B Virus: Challenges of Curative Therapies. *J Virol, 93*(20). doi:10.1128/JVI.01032-19

Liao, Y., Smyth, G. K., & Shi, W. (2014). featureCounts: an efficient general purpose program for assigning sequence reads to genomic features. *Bioinformatics, 30*(7), 923-930. doi:10.1093/bioinformatics/btt656

McKenna, A., Hanna, M., Banks, E., Sivachenko, A., Cibulskis, K., Kernytsky, A., . . . DePristo, M. A. (2010). The Genome Analysis Toolkit: a MapReduce framework for analyzing next-generation DNA sequencing data. *Genome Res, 20*(9), 1297-1303. doi:10.1101/gr.107524.110

Okonechnikov, K., Conesa, A., & Garcia-Alcalde, F. (2016). Qualimap 2: advanced multi-sample quality control for high-throughput sequencing data. *Bioinformatics, 32*(2), 292-294. doi:10.1093/bioinformatics/btv566

Schmieder, R., & Edwards, R. (2011). Quality control and preprocessing of metagenomic datasets. *Bioinformatics, 27*(6), 863-864. doi:10.1093/bioinformatics/btr026

Wei, L., & Ploss, A. (2020). Core components of DNA lagging strand synthesis machinery are essential for hepatitis B virus cccDNA formation. *Nat Microbiol*. doi:10.1038/s41564-020-0678-0
